# Fast Recognition of BCI-Inefficient Users Using Physiological Features from EEG Signals: A Screening Study of Stroke Patients

**DOI:** 10.3389/fnins.2018.00093

**Published:** 2018-02-21

**Authors:** Xiaokang Shu, Shugeng Chen, Lin Yao, Xinjun Sheng, Dingguo Zhang, Ning Jiang, Jie Jia, Xiangyang Zhu

**Affiliations:** ^1^State Key Laboratory of Mechanical System and Vibration, Shanghai Jiao Tong University, Shanghai, China; ^2^Department of Rehabilitation, Huashan Hospital, Shanghai, China; ^3^Department of Systems Design Engineering, Faculty of Engineering, University of Waterloo, Waterloo, ON, Canada

**Keywords:** brain-computer interface (BCI), stroke rehabilitation, motor imagery (MI), sensori-motor rhythm (SMR), BCI-inefficiency

## Abstract

Motor imagery (MI) based brain-computer interface (BCI) has been developed as an alternative therapy for stroke rehabilitation. However, experimental evidence demonstrates that a significant portion (10–50%) of subjects are BCI-inefficient users (accuracy less than 70%). Thus, predicting BCI performance prior to clinical BCI usage would facilitate the selection of suitable end-users and improve the efficiency of stroke rehabilitation. In the current study, we proposed two physiological variables, i.e., laterality index (LI) and cortical activation strength (CAS), to predict MI-BCI performance. Twenty-four stroke patients and 10 healthy subjects were recruited for this study. Each subject was required to perform two blocks of left- and right-hand MI tasks. Linear regression analyses were performed between the BCI accuracies and two physiological predictors. Here, the predictors were calculated from the electroencephalography (EEG) signals during paretic hand MI tasks (5 trials; approximately 1 min). LI values exhibited a statistically significant correlation with two-class BCI (left vs. right) performance (r = −0.732, *p* < 0.001), and CAS values exhibited a statistically significant correlation with brain-switch BCI (task vs. idle) performance (*r* = 0.641, *p* < 0.001). Furthermore, the BCI-inefficient users were successfully recognized with a sensitivity of 88.2% and a specificity of 85.7% in the two-class BCI. The brain-switch BCI achieved a sensitivity of 100.0% and a specificity of 87.5% in the discrimination of BCI-inefficient users. These results demonstrated that the proposed BCI predictors were promising to promote the BCI usage in stroke rehabilitation and contribute to a better understanding of the BCI-inefficiency phenomenon in stroke patients.

## 1. Introduction

Brain-computer interface (BCI) provides a direct communication and control channel between human brain and external devices (Pfurtscheller and Neuper, 2001; Wolpaw et al., 2002). In past decades, various BCI modalities have been developed for different applications (Cincotti et al., 2008; Chaudhary et al., 2016). Among them, motor imagery-based BCI (MI-BCI) is widely used for volitional control by voluntary modulation of sensorimotor rhythm (SMR). Recently, MI-BCI has been proposed as an alternative neural therapy for stroke rehabilitation (Daly and Wolpaw, 2008; Soekadar et al., 2015). It can effectively induce beneficial plastic changes in stroke patients (Shindo et al., 2011).

Although clinical studies have demonstrated the effectiveness of MI-BCI for stroke rehabilitation (Ramos-Murguialday et al., 2012, 2013; Pichiorri et al., 2015), significant variance in the outcomes is noted among different subjects. Different factors may contribute to the variance of rehabilitation outcomes (e.g., lesion type, lesion side, post-stroke time, and BCI performance). Bundy et al. (2017) recently demonstrated that the BCI-based rehabilitation outcome is statistically associated with the BCI decoding accuracy. In detail, patients with higher BCI decoding accuracy achieved better recoveries of motor function after 12 weeks of home-based BCI treatments. Thus, the “BCI-inefficiency” phenomenon (Hammer et al., 2012; Edlinger et al., 2015), also termed as “BCI-illiteracy” problem (Kübler and Müller, 2007), is considered as a critical issue confronting the clinical application of BCI-based rehabilitation. Specifically, approximately 40% of stroke patients can not achieve the critical BCI accuracy level of 70% (Ang and Guan, 2015). The BCI-based rehabilitation therapy is probably invalid in these BCI-inefficient users. Therefore, recognition of BCI-inefficient users may facilitate the practical application of MI-BCI in stroke rehabilitation.

To date, the underlying neural mechanism of the BCI-inefficiency phenomenon remains poorly understood and has attracted extensive interests (Guger et al., 2003, 2009; Vidaurre and Blankertz, 2010). To distinguish BCI-inefficient users more efficiently, a great number of BCI predictors have been identified. Ahn and Jun (2015) reviewed and gathered the existed MI-BCI predictors into four categories, i.e., personal information, psychological, anatomical and physiological factors. Based on the underlying causes and correlates of BCI performance variation, the authors subsequently proposed strategic approaches to address the BCI-inefficiency problem. On the other hand, Kleih and Kübler (2015) focused on the effects of psychological factors on both MI- and P300-BCI performances, and an integrative model of BCI control was suggested to integrate all factors on BCI performances. More recently, Jeunet et al. (2016) investigated the psychological and cognitive factors on MI-BCI performance, and summarized that MI-BCI performance was effected by the user' relationship with technology, attention and spatial abilities.

Psychological factors have been greatly concerned in the previous studies. Burde and Blankertz (2006) found that a person who had more confidence in technology and their own ability achieved better BCI performance. BCI accuracy in that study was significantly correlated with the “locus of control of reinforcement” (*r* = 0.59). Conversely, it has been demonstrated that the fear of BCI-control failure may decrease BCI performance (Witte et al., 2013). Moreover, Vuckovic and Osuagwu (2013) indicated that MI abilities evaluated with questionnaire scores explained up to 53% of the BCI performance from 30 healthy volunteers. Meanwhile, the visual-motor coordination ability has also been shown to be positively correlated with BCI performance (Hammer et al., 2012, 2014). With these mentioned psychological factors, BCI-inefficient users could be easily recognized. It may highly benefit the practical applications of MI-BCI system.

Measurement of physiological features has provided a more objective pathway to predict BCI performance. Blankertz et al. (2010) proposed a physiological BCI predictor that can be determined using only 2 min of EEG signals during resting state. They observed a strong correlation of *r* = 0.53 between resting-state alpha activity and BCI accuracies in 80 BCI-naive participants. Ahn et al. (2013b) demonstrated that high theta and low alpha rhythms during resting state were correlated with poorer BCI performances, whereas higher gamma rhythms in the frontal areas resulted in higher BCI accuracies (Ahn et al., 2013a). Conversely, Bamdadian et al. (2014) proved that higher frontal theta and lower posterior alpha brain activities during rest were correlated with better BCI performance. Furthermore, Grosse-Wentrup et al. (2011) showed that MI-induced SMR features were positively correlated with the frontal and occipital gamma rhythms, and negatively correlated with the centro-parietal gamma rhythms. The same group further demonstrated that resting-state gamma rhythms predicted the single-trial BCI classification accuracy (Grosse-Wentrup and Schölkopf, 2012). More recently, Zhang et al. (2015) demonstrated a close relationship between resting-state brain network and MI-BCI accuracies. On the other hand, anatomical features measured by fMRI were also found to be significantly correlated with MI-BCI accuracy, providing further insight into the mechanism of BCI-inefficiency phenomenon (Halder et al., 2011, 2013; Zhang et al., 2016).

However, all the aforementioned BCI predictors were developed and validated on healthy subjects. To our knowledge, only a few of works have targeted BCI performance variations in patients. Neumann and Birbaumer (2003) explored BCI performance in five severely paralyzed patients. They demonstrated a linear correlation between the decoding accuracies in initial runs (runs 1–31) and later runs (runs 64–94 and 162–191). Similarly, the results from amyotrophic lateral sclerosis (ALS) patients demonstrated a high positive correlation of *r* = 0.87 between the BCI accuracies in the 3rd and 6th sessions (Kübler et al., 2004). Effects of psychological features on BCI performance variations were also investigated in ALS patients (Nijboer et al., 2010). The results showed that motivation factors (i.e., challenge and mastery confidence) of patients were positively related to MI-BCI performance. Nevertheless, a physiological predictor of MI-BCI performance in stroke patients is currently not available.

In this paper, two physiological predictors, i.e., cortical activation strength (CAS) and laterality index (LI), were proposed to recognize BCI-inefficient users in stroke patients by roughly recording of 1-min EEG signals when subjects performed imagined hand movements. We hypothesized that the CAS values from the motor cortex could be used to predict the brain-switch BCI (task vs. idle) accuracies, and the LI values of event-related spectrum perturbation (ERSP) would be feasible in the prediction of two-class BCI (left vs. right) accuracies. The effectiveness of our proposed predictors were experimentally validated across a number of stroke patients.

## 2. Materials and methods

### 2.1. Subjects

The study cohorts consisted of 24 stroke patients (4 females, age of 48 ± 14 years) and 10 healthy subjects (3 females, age of 28 ± 5 years). The patients were recruited from the Rehabilitation Department of Huashan Hospital based on the following inclusion criteria: (1) impairment of unilateral hemisphere with significant motor dysfunction; (2) less than 70 years old; (3) with a normal cognitive function; (4) be able to follow simple verbal commands and freely communicate with experimenters. The exclusion criteria were as follows: (1) unstable medical conditions; (2) do not agree with the informed consent; (3) have the history of seizure, or other conditions with potential influences on the study. The motor and cognitive functions of stroke patients were assessed by the therapists using Fugl-Meyer Assessment for Upper Extremity (FMA-UE, range of 0–66 scores) and Mini-Mental State Examination (MMSE, range of 0–30 scores), separately. Patients with FMA-UE scores > 50 or MMSE scores < 27 were excluded. The characteristics of recruited patients are listed in Table 1. The healthy subjects were recruited from Shanghai Jiao Tong University. They were not affected by any physical or psychological disease and had normal or corrected to normal vision. This study was approved by the ethical committee of Huashan Hospital. All subjects signed informed consent forms in accordance with the Declaration of Helsinki.

**Table 1:** Characteristics of stroke patients.

**Patient**	**Affected hand**	**Time post-stroke (mo)**	**Injury type**	**Lesion site**	**MMSE**	**FMA-UE**
P1	R	2	Ischemia	Cortical	30	37
P2	R	23	Ischemia	Sub-cortical	28	4
P3	R	1	Hemorrhage	Sub-cortical	30	50
P4	R	84	Ischemia	Sub-cortical	29	12
P5	L	20	Ischemia	Sub-cortical	30	24
P6	R	55	Hemorrhage	Cortical	30	6
P7	L	120	Hemorrhage	Cortical	39	6
P8	L	32	Hemorrhage	Sub-cortical	30	25
P9	L	32	Hemorrhage	Sub-cortical	30	7
P10	R	28	Ischemia	Sub-cortical	30	5
P11	L	16	Ischemia	Sub-cortical	30	12
P12	R	35	Hemorrhage	Cortical	30	5
P13	L	34	Hemorrhage	Cortical	30	4
P14	R	12	Hemorrhage	Sub-cortical	30	12
P15	R	5	Ischemia	Sub-cortical	30	38
P16	L	16	Hemorrhage	Cortical	30	22
P17	L	4	Ischemia	Cortical	30	10
P18	R	3	Hemorrhage	Sub-cortical	29	17
P19	L	16	Hemorrhage	Sub-cortical	28	25
P20	R	20	Ischemia	Sub-cortical	27	28
P21	R	1	Ischemia	Cortical	30	10
P22	L	5	Ischemia	Cortical	29	18
P23	L	8	Ischemia	Cortical	30	7
P24	R	10	Ischemia	Cortical	27	36

### 2.2. Experimental paradigm

The experiments in stroke patients were conducted in Huashan Hospital with aides of the therapists, whereas the experiments in healthy subjects were performed in the BioMechatronics and BioRobotics Laboratory at Shanghai Jiao Tong University. During these experiments, subjects were seated in a comfortable chair with hands resting on the armrests. A 23-inch sized screen was set approximately 60 cm in front of subjects to display visual cues. Each subject was required to complete two blocks of hand MI, and a rest as long as their wish was taken between two blocks. Each block consisted of 20 trials for left-hand MI and 20 trials for right-hand MI. The entire experiment totally contained 80 trials and lasted approximately 40 min (including the time for preparation).

**Figure 1:**
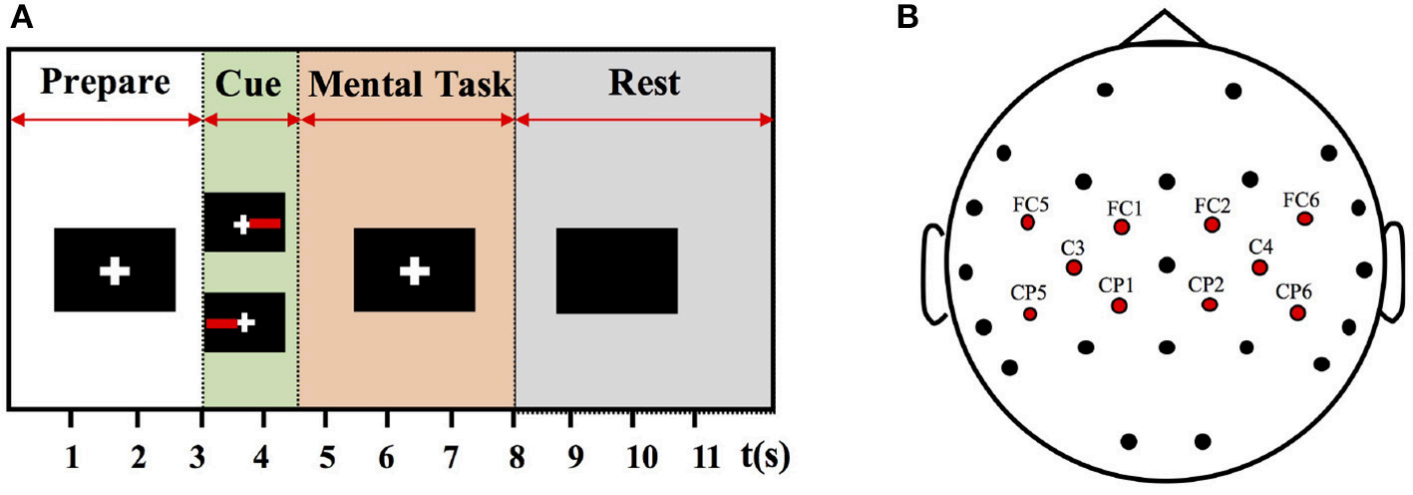
**(A)** Procedure of a single trial. **(B)** Distribution of EEG channels. These channels indicated with red dots are selected for further analysis of motor cortical activations.

The experimental protocol is illustrated in Figure 1A. At the beginning of each trial, there was a white cross appeared on the screen with black background. The white cross lasted about 3 seconds to remind the subject to focus his mind and keep still. A red rectangle was displayed on the left or right side of the cross to indicate a left- or right-hand MI, respectively, and the tasks were in random order. Subjects were required to perform the corresponding hand MI tasks immediately when the cues appeared. The mental tasks were predefined to mentally mimic the clenching movements of indicated hand. Only the red rectangle, not the white cross, disappeared after 1.5 s. The subjects should continue the MI task until the white cross disappeared after 8 s. Then, there was a short break of 6–8 s before the next trial. The idle state was defined at [−4 −1] s prior to task cues and the task state was defined at [1 4] s post task cues. During the whole experiment, all subjects were required to avoid any additional facial or arm muscular movements. The subjects were informed that they can terminate the experimental session at any point without question.

### 2.3. EEG recording

EEG signals of stroke patients were recorded using a BrainAmp amplifier (Brain Products, Gilching, Germany) and 32 channels of active Ag/AgCl electrodes (actiCAP, Brain Products, Germany). The low-pass filter setting was 0–100 Hz with a sampling rate of 200 Hz, and a 50 Hz notch filter was used to diminish power line interference. EEG signals of healthy subjects were recorded using a SynAmps2 system (NeuroScan, U.S.A.) and a quick-cap with 64 Ag/AgCl electrodes. The sampling rate of healthy subjects was 250 Hz and raw data were filtered with an analog bandpass filter from 0.5 to 70 Hz and a notch filter of 50 Hz. The electrodes were placed according to the extended 10–20 system. The ground channel was located on the forehead, and the reference channel was located on the vertex. Impedances of all electrodes were kept below 5 kΩ. Different EEG recording systems were used for healthy subjects and stroke patients, because the experiments of stroke patients were carried out in a clinical setting and only the BrainAmp system was allowed and approved in the ethics. Although 64 channels of EEG signals were recorded from healthy subjects, only 32 channels which had the same locations of electrodes as those of stroke patients were used in further analysis.

### 2.4. Analysis method

To better understand the cortical activation patterns from stroke patients, SMR features with respect to MI tasks were analyzed in the time-frequency and spatial domains. As a variant of event-related desynchronization (ERD), event-related spectral perturbation (ERSP) was used to visualize the spectral power changes corresponding to different motor tasks (Graimann et al., 2002; Makeig et al., 2004). ERSP was generally formulated as

(1) $$ERSP(f,t)=\frac{1}{n}\sum_{k=1}^{n}(F_{k}(f,t{)}^{2})$$

where *n* was the number of trials, and *F*_*k*_(*f, t*) was the spectral estimation of the *k*th trial with frequency = *f* and time = *t*. Short-time Fourier transform (STFT) was applied in time-frequency analysis of EEG data. The analysis was performed with a Hanning-tapered window using EEGLAB (Delorme et al., 2011). Note that the ERSP values were log-transformed prior to further analysis. Since a decrease in band power with respect to the baseline represents cortical activation, smaller ERSP values during MI tasks are correlated with larger cortical activations (Qiu et al., 2016).

To visualize the event-related cortical activations, ERSP values were normalized by subtracting the baseline of *t* = [−2 −1] s for each channel, and averaged over task period *t* = [1 4] s and individual frequency band *f* = [*N* *N*+5] Hz for each subject. The index *N* was the cutoff frequency of individual frequency band, and ranged from 5 to 25 Hz with an interval of 1 Hz. Hence, the individual frequency bands were sub-bands of [5 30] Hz which has covered the theta ([5 7] Hz), alpha ([8 13] Hz), and beta ([14 30] Hz) bands. We selected the individual frequency band [*N* *N*+5] Hz which resulted in the smallest ERSP values at channel C3 and C4. Aimed for BCI prediction, the ERSP values were determined using the first 5 trials of paretic hand MI. For healthy subjects, the individual frequency bands were selected in the same way during non-dominant hand MI. It is worth noting that the selected individual frequency bands maybe not the optimal parameter for BCI classification, however the frequency bands with larger activations are more meaningful for stroke rehabilitation (Johansen-Berg et al., 2002). These parameters were also used to plot the power spatial distributions of different mental tasks with the Fieldtrip toolbox (Oostenveld et al., 2011).

Two indexes were proposed to predict the BCI performances. They were defined as

(2) $$LI=ERSP_{contralateral}-ERSP_{ipsilateral}$$

(3) $$CAS=|ERSP_{contralateral}|+|ERSP_{ipsilateral}|$$

where *ERSP*_*contralateral*_ and *ERSP*_*ipsilateral*_ indicated the averaged ERSP values of the interested electrodes from contralateral and ipsilateral hemispheres, respectively. The channels of interests are presented in Figure 1B with red dots. They are FC5, FC1, C3, CP5, and CP1 in the left hemisphere, and FC2, FC6, C4, CP2, and CP6 in the right hemisphere. The indexes *LI* and *CAS* were calculated from the very first 5 trials or 5 random trials of paretic hand MI tasks. These trials together with other 5 trials of un-paretic hand MI tasks were excluded for BCI decoding. Then, linear regression analyses were performed between the physiological predictors and BCI accuracies. In terms of two-class BCI, activation patterns of different MI tasks play a crucial role in BCI performance, and more lateralized activation patterns are associated with higher BCI accuracies (Kasahara et al., 2015); In terms of brain-switch BCI, activation strength derived from hand MI determines the BCI performance, and higher activation levels are expected to produce better BCI performance. Thus, LI values were expected to be correlated with two-class BCI accuracies, and CAS values were expected to be correlated with brain-switch BCI accuracies. Additionally, in order to obtain a relatively robust regression model, we calculated the Mahalanobis distance to the data center for each patient (Blankertz et al., 2010). A threshold of Mahalanobis distance = 2 was used to reject the outliers.

BCI performances were evaluated using the decoding accuracy between different mental tasks. Two different BCI modalities were involved: (1) left-hand MI vs. right-hand MI (two-class BCI); (2) task state vs. idle state (brain-switch BCI) of paretic hand MI. In this study, common spatial pattern (CSP) (Ramoser et al., 2000) was used for feature extraction, and two pairs of feature patterns were selected for classification. Then the method of linear discriminant analysis (LDA) was employed for discriminating different tasks. The pattern classifications were conducted offline with all 32 channels of EEG signals. EEG features were extracted from the time segment of [1 4] s and individual frequency band of [*N* *N*+5] Hz. A 5 × 5 fold cross validation was performed as follows: (1) 70 trials of MI tasks were randomly permutated and equally divided into five portions, (2) each portion was tested with the classifier which was calibrated using the remaining four portions, (3) this process was repeated 5 times generating 25 decoding accuracies. Then, the averaged classification accuracy and the standard deviation of each patient were used to evaluate the BCI performance.

Recognition of BCI-inefficient users was conducted based on the regression models between predictors (i.e., LI and CAS computed from the very first five trials) and BCI accuracies. Each patient was recognized according to the regression model calibrated with the data from remaining 23 patients. The threshold for BCI-inefficiency was set to 70% for both BCI modalities. The recognition performance was evaluated with sensitivity and specificity, which were defined as

(4) $$Sensitivity=\frac{TP}{TP+FN}$$

(5) $$Specificity=\frac{TN}{TN+FP}$$

where *TP*, *FN*, *TN*, and *FP* represented true positive, false negative, true negative and false positive, respectively. *TP* was the number of BCI-inefficient users who were correctly recognized as BCI-inefficient users, *FN* was the number of BCI-inefficient users who were incorrectly identified as BCI-efficient users, *TN* was the number of BCI-efficient users who were correctly recognized as BCI-efficient users, and *FP* was the number of BCI-efficient users incorrectly predicted to be BCI-inefficient users.

## 3. Results

### 3.1. MI-BCI performance

**Figure 2:**
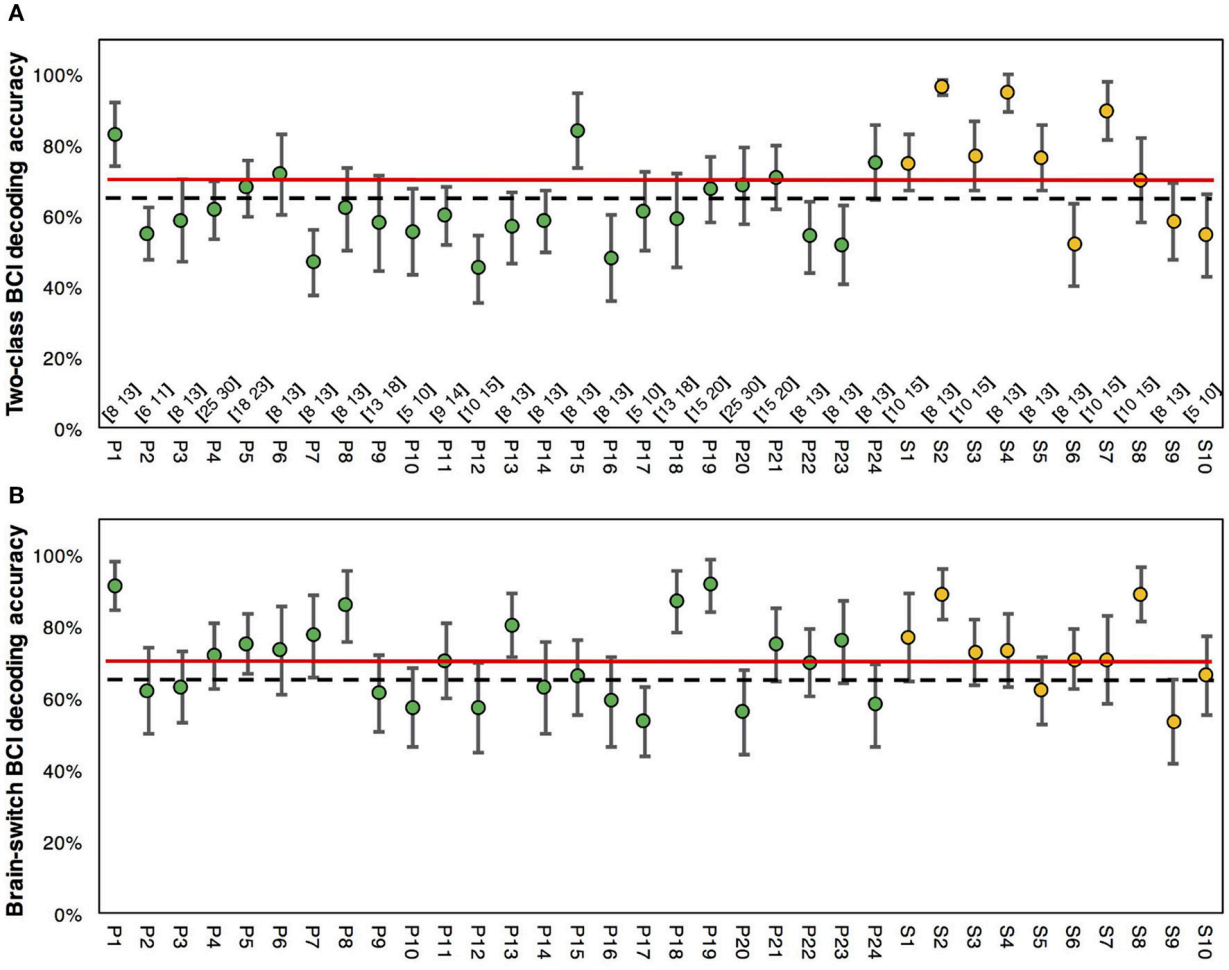
MI-BCI decoding accuracies of stroke patients and healthy subjects. **(A)** Two-class BCI decoding accuracy of all subjects. Selected individual frequency bands are presented on the bottom. **(B)** Brain-switch BCI decoding accuracy of all subjects. The green markers indicate the results of stroke patients, whereas the yellow markers indicate results of healthy subjects. The red lines represent the accuracy level of 70% for BCI-inefficiency, and the black dash lines represent the trial-number corrected chance level of 65% with *p* = 0.05. The error bars represent standard errors.

MI-BCI performances of stroke patients and healthy subjects are presented in Figure 2. In Figure 2A, the averaged two-class BCI decoding accuracy was 61.1 ± 2.0% for stroke patients, and 5 out of 24 patients exceeded the BCI-inefficiency level of 70%. For healthy subjects, the averaged accuracy was 74.3 ± 4.8%, and 7 out of 10 subjects achieved accuracies greater than 70%. Statistical analysis revealed a significant difference (*p* < 0.01, un-paired *t*-test) between these two different populations. Figure 2B presented the results of brain-switch BCI. The averaged accuracy was 70 ± 2.3% for stroke patients and 72.5 ± 3.3% for healthy subjects. No significance (*p* = 0.558, un-paired *t*-test) was found in the inter-group comparison. In addition, 50% (12 out of 24) of stroke patients and 70% (7 out of 10) of healthy subjects exceeded the accuracy threshold of 70% in brain-switch BCI. Interestingly, several patients (P8, P13, P18, P19) with accuracies less than 70% in two-class BCI achieved considerably higher accuracies (>80%) in brain-switch BCI.

According to the BCI decoding accuracy, stroke patients were divided into two groups based on the threshold of 70%. Thus, 12 patients with accuracies above 70% were assigned to the Efficient-Group, and the other 12 patients were assigned to the Inefficient-Group in brain-switch BCI. On the other hand, 5 patients with accuracies above 70% were assigned to the Efficient-Group, and the remaining 16 patients were assigned to the Inefficient-Group in two-class BCI. EEG feature analyses were performed among different groups in two BCI modalities.

### 3.2. Inter-group comparison of EEG features

**Figure 3:**
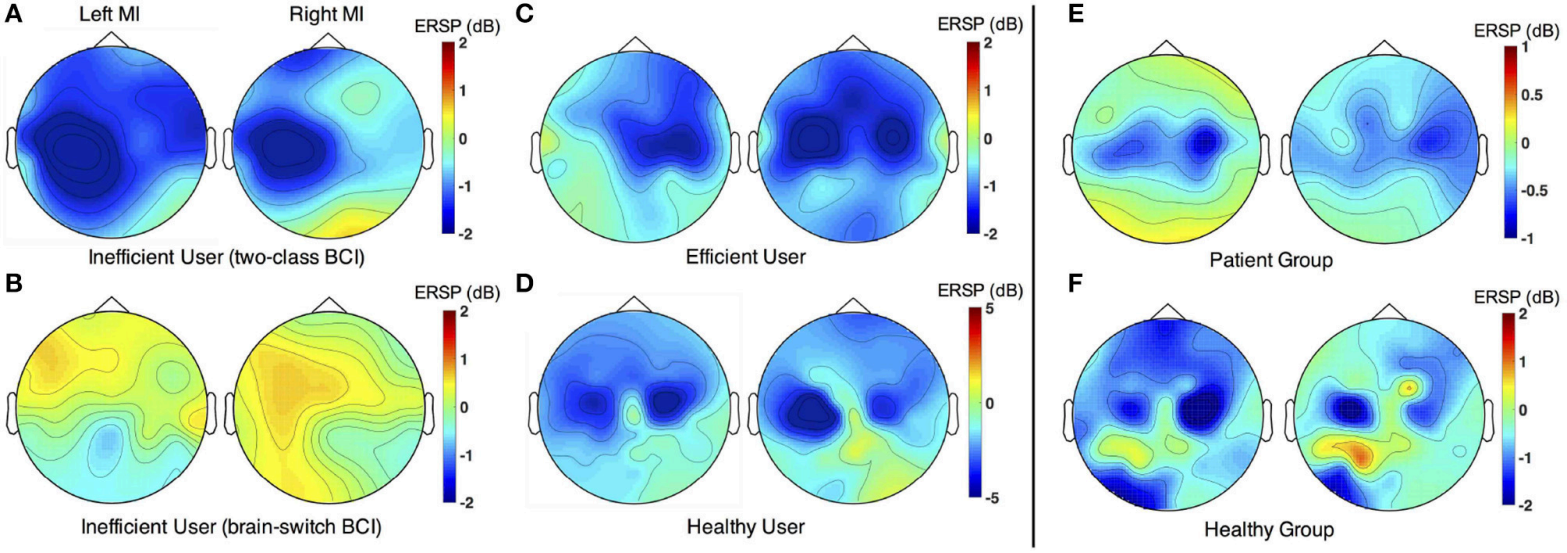
Comparison of ERSP spatial distribution between different MI tasks. In each subplot, the left and right topographic maps are corresponding to left- and right-hand MI, separately. **(A)** ERSP spatial distribution of a representative subject (P8) from the Inefficient-Group in two-class BCI. **(B)** ERSP spatial distribution of a representative subject (P16) from the Inefficient-Group in brain-switch BCI. **(C)** ERSP spatial distribution of a representative subject (P21) from the Efficient-Group in both two-class BCI and brain-switch BCI. **(D)** ERSP spatial distribution of a representative subject (S8) from the Healthy-Group. **(E)** Grand average topographic maps for all stroke patients. ERSP distributions of the patients with paralysis on left side were vertically mirrored in order to illustrate the effect of lesion side on ERSP patterns. Thus, the left and right subplots represent un-paretic and paretic hand MI, respectively. **(F)** Grand average topographic maps for all healthy subjects. The representative subjects in **(A,B)** were patients with paralysis on the left side, whereas the representative subject in **(C)** was a patient with paralysis on the right side. The maps were drawn within the selected individual frequency bands. ERSP values were averaged at [1 4] s and normalized by subtracting the baseline power at [−2 −1] s with respect to the starting cues.

Spatial distributions of ERSP values were compared between different MI tasks. Figure 3A is for a BCI-inefficient user in two-class BCI. Obvious activations were observed during both paretic (left) and un-paretic (right) hand MI. However, the cortical activations during paretic hand MI were ipsilateral to the imagined hand. The activation patterns were similar between paretic and un-paretic hand MI. Figure 3B displays the results of a BCI-inefficient user in brain-switch BCI. No obvious activity was observed during either hand MI. Figure 3C represents a BCI-efficient user in both two-class BCI and brain-switch BCI. The results showed distinct contralateral activations during both paretic (right) hand and un-paretic (left) hand MI. As expected, the healthy subject in Figure 3D exhibited lateralized activation patterns during MI of both hands. Grand average topographic maps for stroke patients and healthy subjects are shown in Figure 3E,F, separately. Both stroke patients and healthy subjects exhibited obvious activations in sensorimotor areas, but the activations of healthy subjects were larger than those of stroke patients. Meanwhile, stroke patients showed larger activations in the ipsilateral hemisphere than contralateral hemisphere during paretic hand MI.

**Figure 4:**
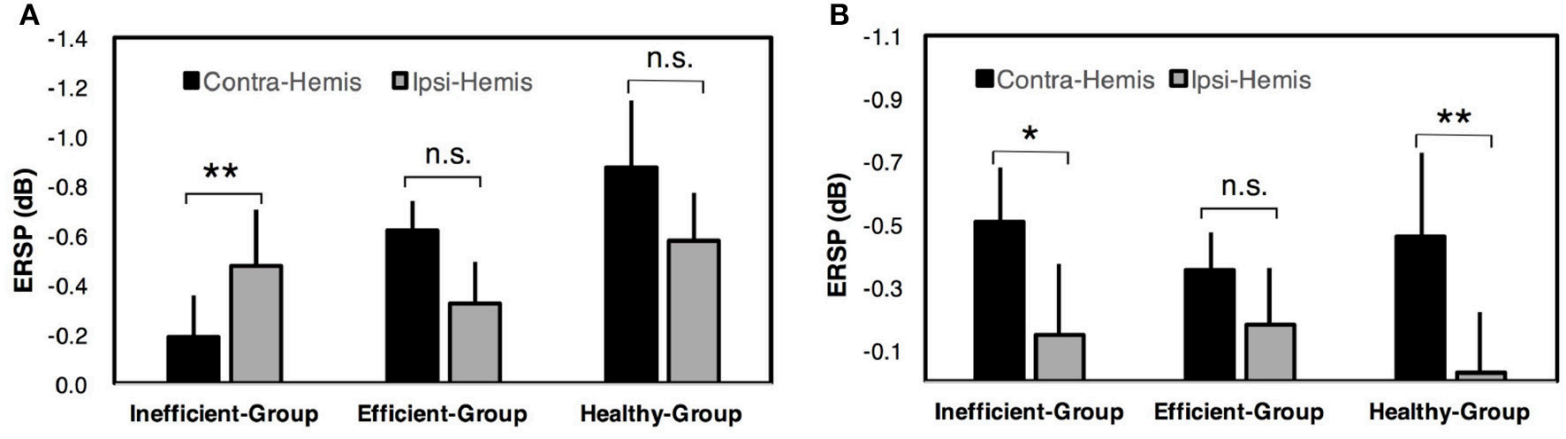
ERSP comparison among different hemispheres in two-class BCI. **(A)** Comparison of ERSP values between contralateral (Contra-Hemis) and ipsilateral hemispheres (Ipsi-Hemis) during paretic/non-dominant hand MI. **(B)** Comparison of ERSP values between contralateral and ipsilateral hemispheres during un-paretic/dominant hand MI. Error bars represent standard errors of the mean ERSP values. ^**^p < 0.01; ^*^p < 0.05; n.s. *p* > 0.05 (paired *t*-test).

For two-class BCI, an inter-group comparison of ERSP values during paretic hand (or healthy subject's non-dominant hand) MI is shown in Figure 4A. The results indicated that the contralateral ERSP values were smaller than the ipsilateral ERSP values for the Efficient-Group and Healthy-Group, but contrast for the Inefficient-Group. In addition, comparison of ERSP values between two hemispheres exhibited a significant difference for the Inefficient-Group (paired *t*-test, *p* < 0.01). The results for un-paretic hand (or healthy subject's dominant hand) MI are presented in Figure 4B. The contralateral motor cortex showed smaller ERSP values compared with the ipsilateral motor cortex for all three different groups. A significant inter-hemispheric difference of ERSP values was observed for the Inefficient-Group (paired *t*-test, *p* < 0.05) and the Healthy-Group (paired *t*-test, *p* < 0.01).

**Figure 5:**
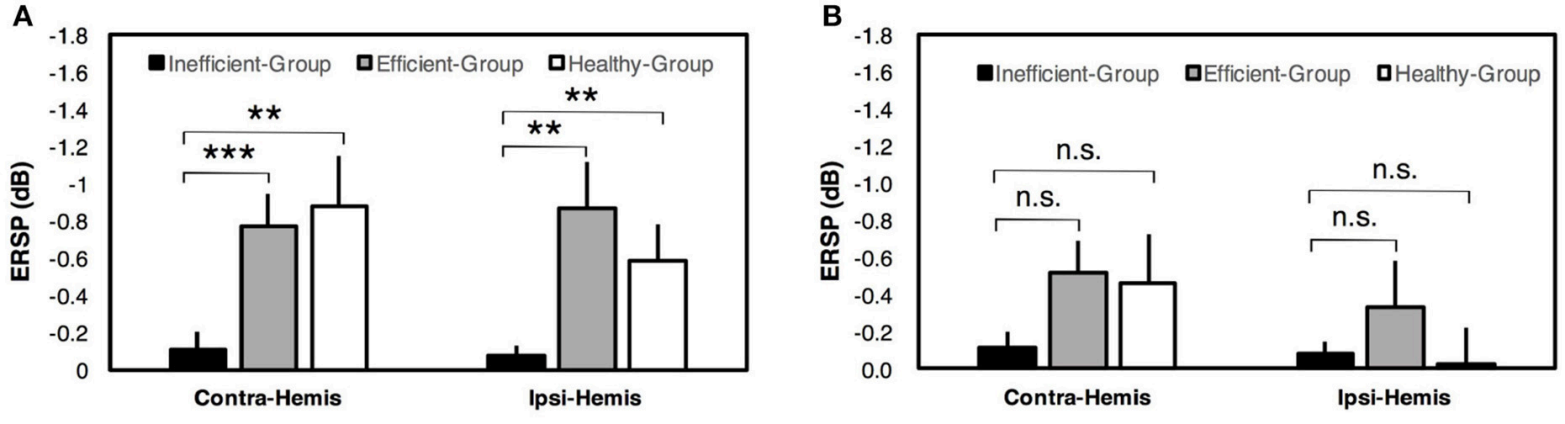
ERSP comparison among different groups in brain-switch BCI. **(A)** Inter-group comparison of ERSP values in contralateral (Contra-Hemis) and ipsilateral hemispheres (Ipsi-Hemis) during paretic/non-dominant hand MI. **(B)** Inter-group comparison of ERSP values in contralateral and ipsilateral hemispheres during un-paretic/dominant hand MI. Error bars represent standard errors of the mean ERSP values. ^***^p < 0.001; ^**^p < 0.01; n.s.*p* > 0.05 (unpaired *t*-test).

For brain-switch BCI, a comparison of ERSP values among different groups is presented in Figure 5. Results for paretic hand (or healthy subject's non-dominant hand) MI are presented in Figure 5A. The Efficient-Group and Healthy-Group showed significantly smaller ERSP values compared with the Inefficient-Group in both contralateral and ipsilateral hemispheres. For un-paretic hand (or healthy subject's dominant hand) MI, smaller ERSP values were observed for the Efficient-Group and Healthy-Group in both hemispheres compared with the Inefficient-Group, but the difference was not significant. Corresponding results are presented in Figure 5B.

### 3.3. Physiological BCI predictors

**Figure 6:**
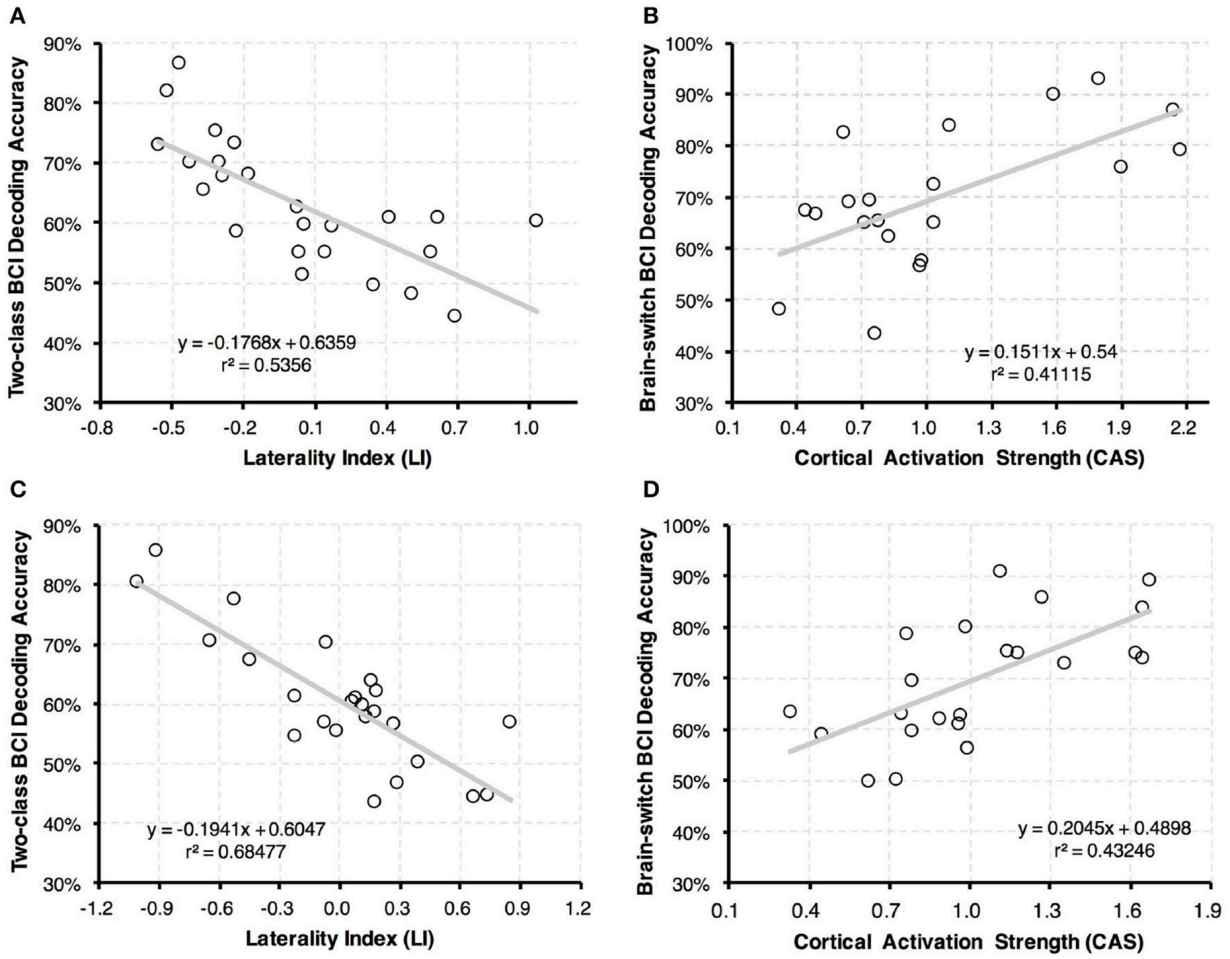
Linear regression results between BCI predictors and classification accuracies. **(A,C)** Linear regression analysis between LI values and two-class BCI classification accuracies. **(B,D)** Linear regression analysis between CAS values and brain-switch BCI classification accuracies. Each dot represents results of one stroke patient. In subplot **(A,B)**, the LI and CAS values were calculated with the first 5 trials of paretic hand MI, and BCI performance was evaluated with the remaining 70 trials of MI tasks (the first 5 trials of un-paretic hand MI were discarded). In subplot **(C,D)**, the LI and CAS values were calculated with 5 randomly selected trials of paretic hand MI, and BCI performance was evaluated with the remaining 70 trials of MI tasks (also 5 trials of un-paretic hand MI were discarded). In **(C,D)**, calculation of both BCI predictors and classification accuracies were repeated for 5 times, and the results were averaged in each patient.

The linear regression analyses between physiological predictors and BCI accuracies are shown in Figure 6. In Figure 6A, a correlation coefficient of *r* = −0.732 was achieved between the LI values and two-class BCI accuracies with *p* < 0.001. This finding demonstrated that two-class BCI accuracy was negatively related to the proposed LI value. Moreover, the CAS values were correlated with brain-switch BCI performance as shown in Figure 6B. A positive correlation with *r* = 0.641 and *p* < 0.001 was found after excluding four outliers. This result indicated that *r*^2^ = 41.1% of brain-switch BCI accuracy variance could be explained with CAS values.

To further validate the efficiency of our proposed method, the BCI predictors were recalculated with 5 randomly selected trials of paretic hand MI. BCI performance was evaluated with the remaining 70 trials (five trials of un-paretic hand MI were discarded). This step was repeated 5 times, and the results of both predictors and BCI accuracies were averaged for each patient. A high correlation coefficient of *r* = −0.828 was achieved between the LI values and two-class BCI accuracies with *p* < 0.001 (see Figure 6C). Similarly, a strong correlation was observed between the CAS values and brain-switch BCI accuracies with *r* = 0.658 and *p* < 0.001 after excluding two outliers (see Figure 6D).

**Figure 7:**
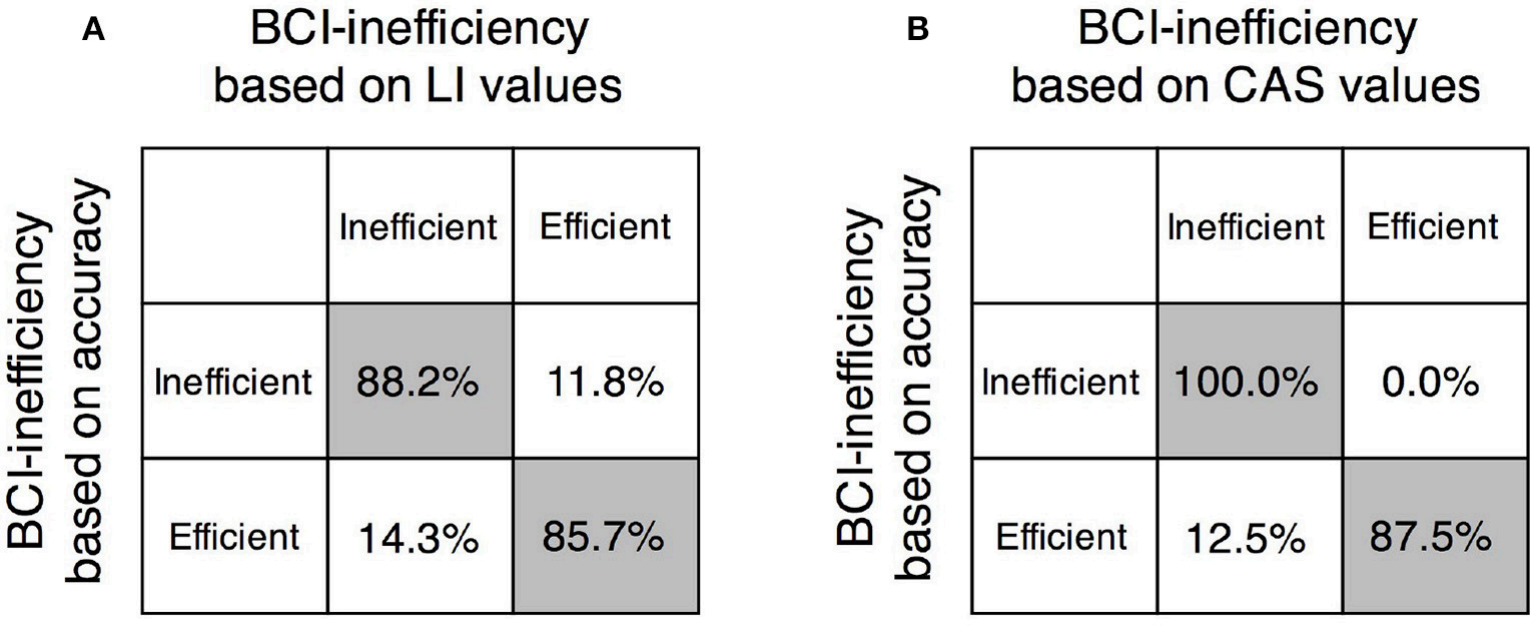
Evaluation of the two proposed physiological predictors. **(A)** Predicting performance in two-class BCI with LI values. **(B)** Predicting performance in brain-switch BCI with CAS values. Sensitivity and specificity for recognition of BCI-inefficient users were 88.2 and 85.7%, respectively, in two-class BCI, and 100.0 and 87.5%, respectively, in brain-switch BCI.

Based on the linear regression model between physiological predictors and BCI accuracies, prediction of BCI-inefficient users was performed in both two-class BCI and brain-switch BCI. The accuracy threshold for BCI-inefficiency was set to 70% for both BCI modalities. As shown in Figure 7A, the BCI-inefficient users were successfully predicted with a sensitivity of 88.2% and a specificity of 85.7% in two-class BCI. The prediction result in brain-switch BCI is shown in Figure 7B, a sensitivity of 100.0% and a specificity of 87.5% were achieved.

## 4. Discussion

The purpose of this study was to investigate the variance of MI-BCI performance among stroke patients, and validate the effectiveness of two physiological predictors on BCI performance. To better understand the neural mechanism of the BCI-inefficiency phenomenon, EEG features were compared among stroke patients and healthy participants.

### Prediction of MI-BCI performance

MI-BCI has been widely researched for stroke rehabilitation. However, a large portion of patients cannot achieve the required BCI accuracy level of 70% (Ang and Guan, 2015). Thus, the selection of suitable BCI users could improve the efficiency of BCI-based rehabilitation. The proposed BCI predictors in this study have demonstrated the potential to solve this problem. According to linear regression results, the recognition of BCI-inefficient users in two-class BCI showed a high sensitivity of 88.2% and a specificity of 85.7%. In addition, the linear regression analysis revealed a strong correlation (*r* = −0.732, *p* < 0.01) between LI values and two-class BCI performance, which indicates that a more lateralized brain pattern is associated with a better two-class BCI performance. This result is in accordance with the findings of Kasahara et al. (2015), in which the BCI accuracy was declared to depend on ERD laterality at 9.5–12.5 Hz. In addition, Zich et al. (2015b) reported that the older adults exhibited less lateralized brain activation patterns and accordingly lower BCI decoding accuracies compared with young adults. However, participants in these studies were healthy subjects, whereas this study targeted at the BCI performance of stroke patients. Therefore, the results in this work have useful implications on clinical applications.

Although great efforts have been made to predict the MI based two-class BCI performance in literatures, the brain-switch BCI strategy is more widely used in stroke rehabilitation (Gomez-Rodriguez et al., 2011; Walter et al., 2012; Ramos-Murguialday et al., 2013). However, to our knowledge, physiological predictors for brain-switch BCI are still limited in patients with paralysis. Nijboer et al. (2010) investigated the influence of psychological state on brain-switch BCI performance. Four patients with amyotrophic lateral sclerosis (ALS) were recruited for 20 sessions of SMR-BCI training, in which patients were required to control a one-dimensional cursor movement with “imagine” and “relaxation” states. The results confirmed that challenge and mastery confidence were positively related to BCI performance, and incompetence fear was negatively related to BCI performance. Unfortunately, due to the small sample size, these psychological parameters did not show a quantitative relation with BCI performance. In the current study, CAS values were proposed as a physiological predictor of brain-switch BCI performance. As shown in Figure 6B, the BCI accuracies were positively correlated with CAS values (*r* = 0.641, *p* < 0.01). The discrimination of BCI-inefficient users in brain-switch BCI exhibited a sensitivity of 100.0% and a specificity of 87.5%.

More importantly, only 1-min EEG signals were required for the discrimination of BCI-inefficient users in this work. In clinical applications, this feature can extensively save time for both patients and therapists. In previous studies, Blankertz et al. (2010) proposed a physiological BCI predictor that can be determined using 2 min of EEG signals at resting state. In the follow-up study, Ahn et al. (2013a) presented a more efficient BCI predictor that can predict the BCI performance with only 1 min of EEG signals. However, the participants in these two studies were all healthy subjects, and the effectiveness of proposed BCI predictors were still unknown on stroke patients. For stroke patients, except for the aforementioned psychological (Burde and Blankertz, 2006; Grosse-Wentrup et al., 2011; Grosse-Wentrup and Schölkopf, 2012; Hammer et al., 2012, 2014; Vuckovic and Osuagwu, 2013), physiological (Blankertz et al., 2010; Ahn et al., 2013a,b; Bamdadian et al., 2014) and anatomical factors (Halder et al., 2011, 2013; Zhang et al., 2016), variations of physiological features caused by pathological factors (i.e., brain hemorrhage, brain ischemia, traumatic brain injury) also play a significant role in BCI performance. Effects of brain damage on cortical activation patterns could be roughly categorized into three cases: (a) patients are unable to voluntarily perform MI tasks or no obvious activation could be observed during mental tasks; (b) motor function of the lesioned cortical area is compensated by the contralesional hemisphere, and MI-induced activations are ipsilateral to the imagined hand; (c) the motor function is not significantly affected by the brain damage and lateralized activation patterns are induced by MI tasks. In the assumption of this paper, case (a) and (b) will decrease the brain-switch BCI and two-class BCI performances, separately, whereas case (c) has no obvious influence on either BCI modality. In the current work, the 1-min EEG signals were used to evaluate the pathological effects on cortical activations which were quantified with the proposed LI and CAS predictors. While EEG signals are commonly regarded as non-stationary, the physiological features caused by pathological factors (i.e., stroke) should be relatively robust, and they are expected to be determined within the 1-min EEG signals (5 trials of paretic hand MI). Thus, our proposed physiological predictors may specifically benefit stroke patients in BCI performance prediction.

### Practical implications on stroke rehabilitation

For those BCI-inefficient users in two-class BCI, the cortical activations corresponding to paretic hand MI were not lateralized to the contralateral side, and cortical activations shifted from the lesioned hemisphere to the healthy hemisphere as demonstrated in Figure 3A. Statistical analysis of cortical activations from the Inefficient-Group further confirmed that paretic hand MI tasks were associated with significantly larger neural activities in the ipsilateral hemisphere (*p* < 0.05), as shown in Figure 4A. This result was consistent with the finding that stroke patients failed to activate their ipsilesional M1 area with paretic hand MI, although the patients reported being able to imagine the movements of either hand (Stinear et al., 2007). Chollet et al. (1991) also demonstrated that the ipsilateral motor areas were substantially recruited during the movements of the affected hand following stroke. This phenomenon is physiologically explained by the fact that the brain has an intrinsic capability to compensate the motor function deficit through neural reorganization (Langhorne et al., 2009). Interestingly, neural compensation does not exist in every stroke patient. For instance, the ERSP values from a BCI-efficient user (Figure 3C) indicated that activations in the contralateral hemisphere were greater than the activities appeared in the ipsilateral hemisphere during the MI of both hands. Therefore, the corresponding patterns were comparable to those of healthy individuals. This difference among stroke patients could be explained that the cerebral network was reorganized during the process of stroke recovery. The motor cortical asymmetry was reduced or even returned to normal as a result of reasonable rehabilitation treatment (Marshall et al., 2000; Takeda et al., 2007).

The current study reveals three implications on the design of BCI system for stroke rehabilitation. First, a well-designed BCI predictor should be applied to identify suitable users with good BCI performance before the BCI treatment. As demonstrated in a clinical study (Bundy et al., 2017), stroke patients with higher BCI accuracies achieved better outcomes after 12 weeks of BCI-based treatments. With the predictors proposed in this study, BCI-efficient users with a mean accuracy of 71.8% for two-class BCI or a mean accuracy of 84.7% for brain-switch BCI could be identified using only 5 trials of paretic hand MI. Those patients classified as BCI-inefficient users should be temporarily eliminated from the BCI-based rehabilitation or turn to other BCI modalities (Dhindsa et al., 2017). Second, as demonstrated in this study, the contralesional hemisphere was associated with significantly larger activities compared with the ipsilesional hemisphere for BCI-inefficient users. Thus, it is plausible that EEG signals from the contralesional hemispheres may achieve a better BCI performance, especially for patients with asymmetrical activation patterns. Bundy et al. (2012) presented an important demonstration in four stroke survivors. In that study, EEG signals from the unaffected hemisphere could be used for successful BCI control. In addition, the same group developed a brain-controlled hand orthosis for motor recovery using neural activities from the ipsilesional hemisphere (Holmes et al., 2012). Hence, signals from the contralesional hemisphere may serve as an alternative neural pathway for BCI control. Finally, the inter-group comparison of EEG features implied that specific treatment to increase ERD laterality, by both increasing the contralateral activations and simultaneously decreasing the ipsilateral activations, may be capable of enhancing the two-class BCI performance. As reported in a previous work, a MI-based training protocol could enhance the ERD laterality within a 3-day training and reasonably improve BCI decoding accuracy (Zich et al., 2015a). Besides, neural modulation techniques, such as transcranial direct current stimulation (tDCS) (Fregni et al., 2005) and transcranial magnetic stimulation (TMS) (Kim et al., 2006) may also help reactivate the lesioned hemisphere of stroke patients.

### Limitations and future work

Several limitations of the current study should be mentioned. In the design of our experiment, we only conduct two blocks of MI tasks, which may be not sufficient to evaluate the BCI performance of recruited patients. In fact, it is a common problem that most stroke patients are with bad physical conditions. A long experiment will cause both physiological and psychological fatigue. Thus, the current experiment was designed to be less than an hour to avoid the effects of fatigue on BCI performance. Meanwhile, different EEG recording systems were used between stroke patients and healthy subjects, which may influence the inter-group comparison of EEG features and BCI performances. In order to minimize this effect, we have taken three steps: (1) keep the electrode impedances of two recording systems at a same range (both below 5 KΩ), (2) chose a same reference electrode (both located on the vertex), (3) only 32 channels with the same locations between two recording systems were used for further analysis. Additionally, individual frequency bands with smallest ERSP values, instead of a fixed band, were used for pattern classification. These frequency bands may not be optimal for discrimination between different mental tasks, but they were more meaningful for stroke rehabilitation (Johansen-Berg et al., 2002). Due to the inter-individual difference of cortical activations, large portions of event-related band powers may fall outside a fixed frequency band which would provide misleading interpretations (Pfurtscheller and Da Silva, 1999). Thus, selection of individual frequency bands using ERSP values was expected to avoid this problem arising with fixed frequency bands. Furthermore, BCI training effects were not considered here. As reported in a previous work (Ono et al., 2013), ERD values were enhanced after a 5-day MI training with visual feedbacks. Accordingly, the BCI performance was significantly improved. Therefore, efficiency of our proposed BCI predictors in well-trained subjects needs to be investigated in future works.

## 5. Conclusion

In current study, we proposed two physiological indexes (i.e., LI and CAS) to predict BCI performance. These predictors exhibited a linear correlation with BCI performance of stroke patients. BCI-inefficient users could be successfully recognized with a high sensitivity and specificity using only five trials of paretic hand MI (approximately 1 min). Inter-group comparison of physiological features showed significant differences between BCI-inefficient and BCI-efficient users. It also demonstrated that BCI-inefficient users were with abnormal brain activation patterns, which were significantly different from that of healthy subjects. This work not only demonstrates an efficient way to recognize BCI-inefficient users, but also provides a further insight into the BCI-inefficiency phenomenon in stroke patients.

## Author contributions

XShu and SC conceived and designed the experiment paradigm. SC, XShu, and LY performed the experiments. XShu analyzed the data and wrote the manuscript. XShe, DZ, NJ, JJ, and XZ reviewed and edited the manuscript. All the authors read and approved the manuscript.

### Conflict of interest statement

The authors declare that the research was conducted in the absence of any commercial or financial relationships that could be construed as a potential conflict of interest.
